# Toward a New Generation of Bio-Scaffolds for Neural Tissue Engineering: Challenges and Perspectives

**DOI:** 10.3390/pharmaceutics15061750

**Published:** 2023-06-16

**Authors:** Francisca Villanueva-Flores, Igor Garcia-Atutxa, Arturo Santos, Juan Armendariz-Borunda

**Affiliations:** 1 Escuela de Medicina y Ciencias de la Salud, Tecnologico de Monterrey, Campus Chihuahua, Av. Heroico Colegio Militar 4700, Nombre de Dios, Chihuahua 31300, Chihuahua, Mexico; 2 Máster en Bioinformática y Bioestadística, Universitat Oberta de Catalunya, Rambla del Poblenou, 156, 08018 Barcelona, Spain; igarcia8@uoc.edu; 3 Escuela de Medicina y Ciencias de la Salud, Tecnologico de Monterrey, Campus Guadalajara, Av. Gral Ramón Corona No 2514, Colonia Nuevo México, Zapopan 45201, Jalisco, Mexico; arturo.santos@tec.mx (A.S.); juan.armendariz.borunda@tec.mx (J.A.-B.); 4 Instituto de Biología Molecular en Medicina y Terapia Génica, Centro Universitario de Ciencias de la Salud, Universidad de Guadalajara, Sierra Mojada 950, Independencia Oriente, Guadalajara 44340, Jalisco, Mexico

**Keywords:** neural tissue engineering, scaffold, hydrogel, nanotechnology, controlled drug delivery

## Abstract

Neural tissue engineering presents a compelling technological breakthrough in restoring brain function, holding immense promise. However, the quest to develop implantable scaffolds for neural culture that fulfill all necessary criteria poses a remarkable challenge for material science. These materials must possess a host of desirable characteristics, including support for cellular survival, proliferation, and neuronal migration and the minimization of inflammatory responses. Moreover, they should facilitate electrochemical cell communication, display mechanical properties akin to the brain, emulate the intricate architecture of the extracellular matrix, and ideally allow the controlled release of substances. This comprehensive review delves into the primary requisites, limitations, and prospective avenues for scaffold design in brain tissue engineering. By offering a panoramic overview, our work aims to serve as an essential resource, guiding the creation of materials endowed with bio-mimetic properties, ultimately revolutionizing the treatment of neurological disorders by developing brain-implantable scaffolds.

## 1. Introduction

Neurological disorders (ND) are one of the primary human disabilities. ND incidence is expected to increase with the aging population. Conventional pharmacological treatments and surgeries focus on reducing symptoms and palliative care, but not on trying to reverse the illness. The main challenges in treating ND are (a) the absence of structural support to permit a repopulation of the lesion cavity and, (b) the heterogeneity of the particularities of each medical case. For example, patients who have suffered from strokes show vast heterogeneity in both brain and behavioral changes, increasing the difficulty of developing effective neurorehabilitation strategies. In this context, it is necessary to generate medical procedures oriented to personalized medicine [1,2].

Tissue engineering has been proposed as a novel alternative for integrating biological elements with materials used in damaged tissue restoration. Neural tissue engineering is primarily a search for strategies to eliminate inflammation and fibrosis upon implantation of foreign materials able to serve as a scaffold for cellular growth. Scaffolds should imitate the natural extracellular matrix to provide a biomimetic environment for neural development. Moreover, scaffolds must retain their structural integrity and stability during the implantation surgery [3,4].

The ideal scaffold for neural tissue engineering should have the following characteristics: (a) lack of toxicity, (b) allows survival, proliferation, and neuronal migration, (c) favors electrochemical cell communication, (d) shows similar mechanical properties to the brain, and ideally, (e) has the ability to release substances in a controlled manner [5,6].

Many scaffolds have been successfully fabricated using various materials and techniques. One of the most promising materials is based on hydrogels, due to their high-water content that mimics soft tissues. Hydrogels are composed of polyelectrolytes able to slow down the release of oppositely charged small molecules, burst out of the cargo, and change their internal network as a response to external stimuli, i.e., changes involving temperature, pH, ultrasound, glucose, or urea concentrations. This class of hydrogels is known as “smart hydrogels” and has significantly impacted the design of materials for tissue engineering. These materials allow the customization of implants according to the needs of each patient [7,8].

Hyaluronic acid (HA) is an example of a widely studied natural polymer that increases the survival rate of dopaminergic neurons and stem cells. HA has been proposed as a promising material for Parkinson’s disease treatment and other therapies for cell replacement [9,10,11]. Nowadays, many natural and synthetic hydrogels are being tested in preclinical trials for peripheral nerve regeneration, with the capacity to release drugs in a controlled manner, expanding their applications [12]. Only collagen has shown satisfactory results in clinical trials for nerve regeneration, but none for brain implantation [13,14].

Some significant drawbacks of hydrogels are their poor mechanical properties, lack of roughness, and high impedance. These are important parameters for adequate neuronal development. Thus, many strategies have been explored to improve hydrogels’ mechanical properties, such as adding carbon nanotubes (CNT) and graphene. These modifications have also enhanced conductivity, making neuronal electrical stimulation possible [15,16,17,18,19]. Unfortunately, many authors have reported the toxicity of carbon derivatives, which lead to inflammation, fibrosis, lung cancer, fetal malformations, oxidative stress, and DNA damage, among others. Other works have explored the use of conductive polymers, but their safety has not yet been established [20,21,22].

## 2. The Challenge of Designing Materials for Neuron Tissue Engineering

The Food and Drug Administration (FDA) has only approved nerve conduits for peripheral nerve repair, while translational products addressing more complex neurological issues are minimal. Injuries in the brain often lead to poor prognoses because of its inability to self-repair. Therefore, developing materials for neural tissue engineering is a major challenge, due to the difficult restoration of functional connectivity between various axons, neural circuits, and non-neuronal cells [23].

A promising strategy for neural tissue restoration is the design of scaffolds that provide a suitable environment for mammalian cell growth and, eventually, develop into a platform for the replacement of the damaged tissue. Scaffolds may have different configurations and elements that together act as templates where cells can proliferate or serve as chemical or physical cell stimulators [24]. Overall, all the physicochemical properties of the scaffold determine how cells interact with the material and ultimately determine the success or failure of the implant [25].

The following sections address the main requirements of materials for brain tissue engineering.

## 3. Main Requirements for Scaffolds for Brain Tissue Engineering

**Figure 1:**
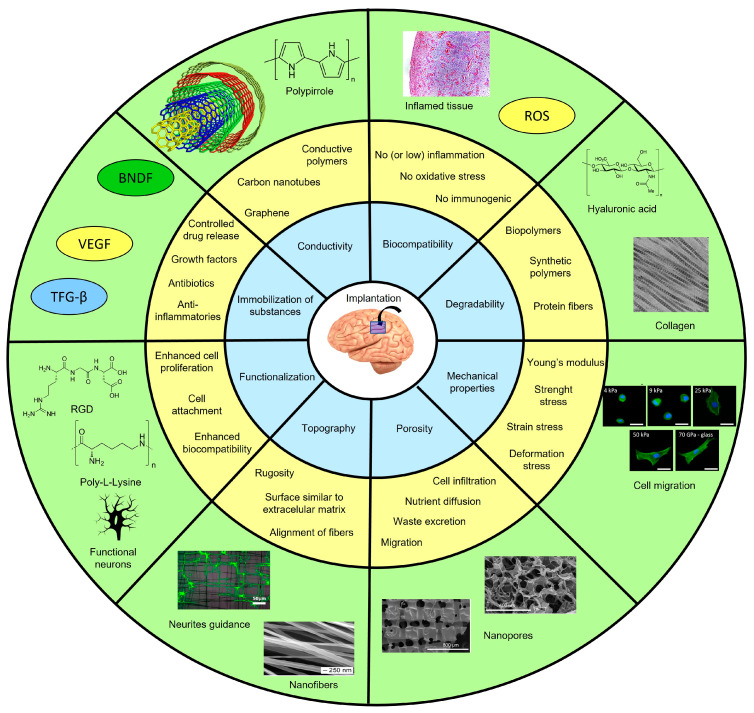
Parameters and strategies for designing scaffolds for brain tissue engineering. In the blue circle, the main parameters to be considered in the design of scaffolds are addressed. The main strategies to cover each parameter are described in the yellow circle. In the green circle, some examples are presented. BNDF: brain-derived *neurotrophic* factor; VEGF: vascular endothelial growth factor; TFG-β: transforming growth factor-beta; ROS: reactive oxygen species; RGD: arginine-glycine-aspartate.

Scaffolds for tissue engineering offer a vast array of possibilities regarding materials and techniques, tailored to the specific needs of the target tissue. Certain key characteristics are crucial for the brain or any other tissue. Biocompatibility and mechanical properties compatible with the target tissue are fundamental for success. In the case of brain scaffolds, additional factors come into play. An optimal topography that promotes neuron attachment, a surface that can be finely tuned, and the ability to immobilize substances within the material core (think antibiotics, anti-inflammatory drugs, growth factors, and neurotrophic factors) are essential. Porosity is also crucial, as it allows for ion exchange and the movement of substances. Moreover, brain scaffolds must minimize microglia activation, ensuring a favorable environment for neuronal growth. Ideally, these scaffolds possess electrical conductivity, which facilitates interneuron communication. To summarize, Figure 1 presents the main parameters to consider when designing scaffolds for brain tissue engineering, serving as a valuable guide in this exciting field.

### 3.1. Biocompatibility and Biodegradability

The most important and obvious characteristics of all scaffolds for tissue engineering are biocompatibility and safety. At the cellular level, the scaffold should allow cell attachment, migration, and proliferation, to favor cells, and the scaffold gets fused to form a functional bio-scaffold. On the other hand, at the tissular level, the scaffold should not be immunogenic to be accepted by the body, and, importantly, it should not be fibrogenic [26].

Many materials have been demonstrated to be biocompatible in the body but fail when they are implanted in the brain. The reason for brain rejection is the brain’s privileged immune response. Unlike the rest of the body, the brain is protected by the blood–brain barrier, a highly selective barrier that allows the passing of just certain substances (water, oxygen, carbon dioxide, etc.) and avoids others (bacteria, viruses, some drugs, etc.). Moreover, the brain lacks lymphatic vessels and antigen-presenting cells. In the brain, foreign substance recognition occurs with the activation of microglia. Activated microglia has two states: (a) the M1 state (proinflammatory), where peripheral leukocytes are infiltrated into the tissue to combat and eliminate infection or injury, and (b) the M2 state (anti-inflammatory), where anti-inflammatory cytokines facilitate phagocytosis of cellular debris and promote the extracellular matrix restoring and tissue repair [27].

The study of brain immune response against intracranially implanted devices has gained attention. After implantation, microglia are immediately activated. Microglia extend their processes to the implant surface (around 130 µm). Twenty-four hours post-implantation, the implanted device is fully surrounded by activated microglia, forming a thin cell capsule. The cellular capsule may limit the ionic exchange and interferes with neuronal communication. During the first week post-implantation, astrocytes are fully activated, and two or three weeks later, astrocytes form a sheath around microglia. Four weeks after implantation, glial cells form tight junctions with each other that limit ion and neurotransmitter diffusion, which ultimately leads to neuronal death and neurite degeneration (approximately 150 µm in radius) [28,29,30].

**Figure 2:**
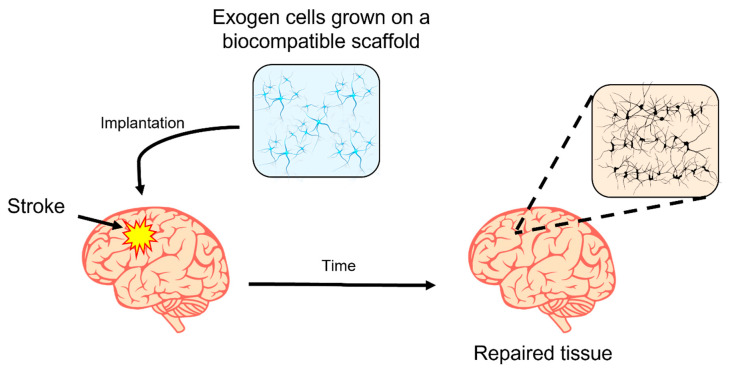
Simplified scheme of an implanted hydrogel used as a platform to establish novel neuronal connections. The hydrogel is a platform for exogen cell growth that establishes novel neuronal connections with the host cells. The cell patch remains in the repaired tissue zone.

Biodegradable implantable devices can reduce potential immunological side effects [31]. Natural hydrogels are the most studied materials for scaffold design that offer mechanical support for neuron growth [32]. A simplified scheme of an implanted hydrogel used as a platform for exogen cell culture and the process for restoring the neuronal connection between exogen and host cells is shown in Figure 2. After a while, it is expected that the brain implant is fully degraded, and a patch of cells establish novel connections restoring the natural function.

The possible toxic effects of the degradation subproducts are often neglected in the literature. Further research should be conducted to study their side effects in vivo in the long-term.

### 3.2. Mechanical Properties

Scaffolds for tissue engineering should be compatible with the target tissue, both at cellular and tissular levels. At the cellular level, adherent cells apply contractile forces on the material and can sense material hardness. Otherwise, variations in hydrogel stiffness, density, composition, orientation, and viscoelastic characteristics all affect cell activity and phenotypes such as morphology, spreading, genetic regulation, axonal development, and cell differentiation. At the tissular level, the scaffolds’ mechanical properties should be like the anatomic site for implantation to minimize friction and be strong enough to allow surgical manipulation [33,34,35].

Robinson et al. (2019) have recently reported a practical and non-destructive method for acquiring the elastic modulus of fibrin using a modified Hertz model for thin films. This method can be applied to characterize the mechanical properties of engineered neural tissues [36]. The study of the scaffolds’ mechanical properties on the neuronal phenotype may be exploited in the design of bioengineered scaffolds that promote nerve regeneration upon injury [37,38].

**Figure 3:**
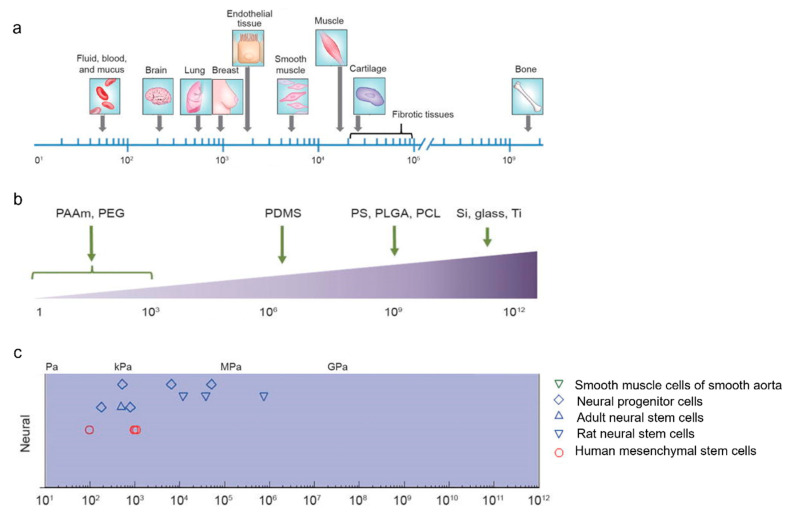
Mechanical properties of the brain and various other materials for neural tissue engineering. Comparing Young’s moduli, we see a differentiation between images located in letters (**a**) brain and other soft tissues, and (**b**) materials applied in neuronal cell culture. The range of Young’s moduli for materials suitable for cultivating neural cells is in image (**c**). PAAm: polyacrylamide, PEG: poly(ethylene glycol), PDMS: poly(dymethylsiloxane), PS: polyestyrene, PLGA: poly(lactic-co-glycolic acid), PCL: poly ɛ-caprolactone, Si: silicon, Ti: Titanium. Modified from [40].

The brain is the softest organ in the body. Designing a material imitating the mechanical properties of the brain and, at the same time, possessing enough hardness to allow cell attachment, is an additional challenge. A comparison between brain stiffness and other tissues and substrates for brain tissue engineering is shown in Figure 3. As can be seen, the brain is barely 0.1–0.3 kPa in stiffness, which is the lowest value in comparison to lung and breast tissue, endothelium, and soft muscle (Figure 3a) [39]. Some of the most-studied polymers for tissue engineering, because of their similar mechanical properties to soft tissues, are polyacrylamide (PAAm), poly(ethylene glycol) (PEG), polydimethylsiloxane (PDMS), polystyrene (PS), poly(lactic-co-glycolic acid) (PLGA), and poly-ε-caprolactone (PCL) (Figure 3b). These materials have been applied as substrates for the culture of various cells such as neurons, adult stem cells, glial cells, rat neural stem cells, and human mesenchymal stem cells, among others [40,41].

Neurons migrate, proliferate, extend their neurites, and develop actin filaments, both soft (0.1 kPa) and hard (GPa) substrates. In contrast, astrocytes proliferate on hard substrates preferentially. It has been observed that co-cultures of neurons and astrocytes growing on soft substrates favor an increased growth of neurons instead of astrocytes. A differential growth may be an advantage for a brain implant because neuronal regeneration is privileged. Otherwise, the development of the glial scar can be decreased [42]. Further research should be conducted to explore the preferential growth of certain cell lineages in co-cultures using scaffolds of various stiffnesses.

### 3.3. Topography

As was previously discussed, neurons are very sensitive to substrate topography. Nanostructured surfaces that imitate extracellular matrix architecture support cell spreading, proliferation, attachment, neurite extension and branching, migration, and the transmission of electrical signals. Moreover, topography influences neural stem cell differentiation [43,44].

Neurons utilize filopodia as their cellular protrusion organelles and depend on specific integrin-mediated adhesions to the local extracellular matrix for guidance in their pathfinding [45]. Recently, a universal mechanism has been proposed for cell alignment in response to substrate topography. This hypothesis indicates that cells may interact with a scaffold through specific surface ligands called focal adhesions. Focal adhesions and actin stress fibers determine cell attachment and spread, through anisotropic force generation, cellular elongation, and alignment. In other words, the control of contact guidance is influenced by a balance of cell-substratum and cell–cell interactions, modulated by cell phenotype-specific cytoskeletal arrangements [46].

The current design of scaffolds for brain tissue engineering is focused on mimicking the natural extracellular matrix topography. Various techniques have been used to frame high-resolution reliefs, to study the cell response to nano topography [47]. Nanofibers and aligned carbon nanotubes enhance biocompatibility, neurite extension, direction, and the branching of neurites, and the alignment of glial cells. In addition, fiber diameter also influences cell differentiation, as has been observed in neural stem cell culture on aligned fibers of poly(lactic) acid (PLLA) of 250 nm in diameter. In contrast, neural stem cells remain undifferentiated when grown on aligned PLLA fibers of different diameters. Cells can grow independently from the fiber’s diameter, but surprisingly, fibers of 250 nm in diameter trigger cell differentiation. Cells have been observed to suffer no changes when grown on aligned PLLA nanofibers of 1.25 µm in diameter [48,49,50,51,52,53,54,55].

Neurons have been cultured on isotropic surfaces (materials with a homogeneous distribution of particles on a solid substrate). Surfaces were built with pillars and holes at the nanometric and micrometric scale in aligned patterns. Pillars of 2 µm were separated by holes of 1.5 µm in diameter. Neuron growth alignment was lost when pillars were separated by more than 3 µm or placed on plane substrates. The study of contact guidance is key to many potential biomedical applications for future brain implants, with improved neurite outgrowth [56,57,58,59].

In a different study, [60] reported that mouse neuroblastoma cells (Neuro2A), grown on a nanostructured silicon substrate, can conduct information 3 to 4 times more efficiently compared to random networks on flat surfaces after 11 days after seeding [36,60]. Further research should be conducted to understand, at the molecular level, the influence of topography in neuronal development, to fabricate better scaffolds for brain tissue engineering. It has been proposed that energetic interactions at the cell–nanomaterial interface favor the synergy between the substrate topography and the cell biochemical signals. Together, they allow the orientation of dendrites and even accelerate neuronal development by up to 1.54 times [47,61,62,63].

Current approaches to designing materials for brain tissue engineering should consider the topography of scaffolds to provide a nano-structured or micro-structured environment to provide growth guidance, control cell differentiation, and favor the integration of the regenerated tissue. Nanostructures direct the extension of axons and induce unique branching morphologies [64]. It was recently observed that the implantation of cultured neuroglia cells into an aligned scaffold of PCL fibers improves sciatic nerve regeneration in rats [65]. The underlined mechanisms of how neurons respond to different nano-topographies and micro-topographies remain unknown. Further research should be conducted to explore novel neural guides and their effect on physiology to create better scaffolds for brain tissue engineering.

An interesting example of the application of the influence of 3D micro morphologies on neural culture is a technology called NeuroGrid^®^, (Silicon Group, Stanford, CA, USA) [66]. NeuroGrid^®^ is a scaffold with defined porous and non-porous regions in its structure that can be used to transfer and manipulate organoids for analytical purposes. NeuroGrid^®^ geometries were inspired by the size of neuronal fiber bundles, (approximately 500 µm) to guide the connectivity between 3D neuronal cell clusters, resulting in a useful tool to create more uniform spheroids with defined diameters. Moreover, NeuroGrid^®^ was able to connect a multi-electrode array (MEA) system to record neuronal activity. This tool can potentially revolutionize how we approach new methodologies to understand neural connectivity to neural tissue engineering [66].

### 3.4. Porosity

Interconnected porous hydrogels exhibit favorable cellular responses compared with traditional non-porous materials [67]. Pore size determines the diffusion rate of nutrients and waste removal. A substrate with an appropriate pore size, facilitates cell seeding, cell penetration, oxygen diffusion, and distribution in the scaffolds. In addition, pore size strongly influences cell adhesion, cell-to-cell interaction, and spreading. Neurons can sense the scaffold surface. Focal adhesions regulate signaling complexes and integrin function and initiate a signaling cascade that stimulates cell proliferation and differentiation [68,69].

Electrospinning has emerged as a promising method for designing materials with controlled porosity. Electrospinning is a versatile, efficient, cheap, and reproducible technique to produce extremely interconnected thin fibers and highly porous microstructures by applying an electrostatic force to a given solution. Many scientific articles report electrospinning as a tool for designing innovative neural scaffolds. Along with these lines, using electrospinning, Moztarzadeh and Sadeghi (2016), generated hyaluronic acid and polycaprolactone nanofibers of various porosities. SH-SY5Y, human neuroblastoma cells, were cultured on the scaffold. The authors found that there is an ideal porosity that facilitates neuronal growth. These results highlight porosity’s importance in eliciting an adequate neuronal growth environment. Moreover, it has been observed that porous scaffolds with anisotropic structures can also be produced to guide cellular proliferation [70]. According to a number of authors, the ideal pore size for neuron culture is around 95–150 µm [67,71,72,73].

Although pore volume can be easily determined using the BET method, based on N_2_ adsorption isotherms using a surface area analyzer, BET analysis requires samples to be completely dried. The drying process alters the internal structure and does not necessarily reveal the real porosity for in vivo applications [74]. Atomic force microscopy (AFM) is one of the microscopic techniques with the highest resolution, that can be applied to characterizing morphological surfaces. The method oscillates ever near the sample surface to form a capillary neck between the tip and the sample, leading to hysteresis in the force-distance curve. Unlike BET, wet samples can be analyzed in AFM more accurately to real-life conditions [75].

Studies on the influence of scaffolds’ pore size are scarce in the literature. Further research is needed to understand the fluid mechanics inside the network to optimize the pore size, guarantee an adequate flux of nutrients and waste removal, and optimize cell attachment.

### 3.5. Immobilization of Active Substances

Immobilizing active substances has resulted in a good strategy to improve host tissue integration with the brain implant, enhance biocompatibility, and provide additional functionality for the scaffold [76,77,78].

Hydrogels usually present low interfacial tension. Thus, they often require the immobilization of substances to support cell attachment. For example, collagen or HA do not require additional functionalization because they naturally possess peptide ligands of type RGD that mimics key biochemical and mechanical features of the brain’s matrix [79]. Synthetic scaffolds are typically covered with poly-L-lysin, fibronectin, gelatin, laminin, collagen, and peptides such as RGD, IKVAV, GRGDS, mi- GDPGYIGSR, and mi-GQASSIKVA, among others [80,81,82,83,84].

The immobilization of growth factors is a successful strategy to enhance scaffold biocompatibility. Three-dimensional cultures carried on scaffolds, with sustained growth factor release, are considered more likely to succeed than conventional methods [85]. A neuronal growth factor is any substance that influences the growth of neurons, and they are also involved in tissue repair and vascularization in such a way that they are very interesting substances to help the integration of the implant with the host tissue [86,87]. Some examples of immobilized growth factors in scaffolds for neural tissue engineering and their advantages are listed in Table 1.

The controlled release of bioactive substances in brain implantable devices primarily relies on simple diffusion, which is regulated by adjusting the cross-linking degree of biopolymer-based scaffolds. However, there is an intriguing opportunity to explore the use of conductive materials and harness the natural electrical activity in the brain. Additionally, investigating the application of “molecular switches” holds promise for modulating the release rate of bioactive agents. It is important to note that, as of what is currently known, these strategies lack in vivo evidence of brain-implanted hydrogels with immobilized bioactives, and further research is needed to evaluate their potential long-term toxicity.

### 3.6. Conductivity

Conductive materials are proposed as promising scaffolds for electrical stimulation. The effects of electrical stimulation have been widely studied. Electrical stimulation enhances axonal regeneration and catecholamine release [96,97,98]. Electrical stimulation is an FDA-approved procedure for Parkinson’s disease treatment, obsessive compulsive disorder, and depression [99,100,101,102]. Moreover, conductive polymers offer new approaches to the functional recovery of the post-stroke brain [103,104].

Technological advances in electrical recording and electrical stimulation have contributed to the understanding of neural networks and enabled individual neuron activation [105]. Unlike in past decades, up-to-date technology can perform electrical stimulation with high spatial accuracy [106].

Carbon-derived materials and conductive polymers have gained significant attention in the development of scaffolds for neuron electrical stimulation. As a result of electrical stimulation, neurons differentiate and develop longer neurites [107,108,109,110]. Carbon nanotubes (CNT) and graphene have been applied to develop conductive scaffolds for neural tissue engineering. Neurons were found to be able to form tight contacts between the proximal and distal compartments and showed increased synaptic frequencies when grown on carbon-derived substrates [111,112,113]. On the other hand, neurons have also shown increased cytotoxicity due to the oxidative stress caused by carbon nanotubes and graphene [114,115,116]. In murine models, carbon-derived materials have also triggered inflammation, fibrosis, lung cancer, fetal malformations, and DNA damage. Cytotoxicity has limited carbon-derivatives in clinical applications [117,118].

Many conductive polymers have been studied to develop materials for neuronal scaffolds and potential electrical stimulators. Conductive polymers allow the direct transfer of electrical, electrochemical, and electromechanical stimuli to cells. Conductive polymers possess π-conjugated electrons in their unsaturated bonds for them to be able to move, so that the π-electrons can open electrical paths. Some examples of conductive polymers applied in neuronal scaffolds are polypyrrole (Ppy) [119,120], polyaniline (PANi) [121,122], poly(3,4-ethylene dioxythiophene) (PEDOT) [123,124], and polythiophene [125,126].

Ppy is a biocompatible polymer that enables neuronal reconnection. Ppy provides a neuroprotective environment, can release growth factors, and enables electrical stimulation, which favors dendrite growth and neuronal stem cell differentiation [127]. However, Ppy is very difficult to solubilize once synthesized, in addition to being mechanically rigid and brittle, which makes it impractical for surgical procedures [54].

PANi, is another biocompatible polymer with neuronal cultures. It is a low-cost and chemically stable material and has the potential to demonstrate electroactive behavior when doped with acids, whereas, in the presence of a base, its electroactive characteristics deteriorate. However, it has been observed that using PANi induces chronic inflammation in rats [128]. The toxic effects of implanted PANi-based materials should be studied in more detail.

PEDOT has been applied in the construction of neuronal culture scaffolds, releasing growth factors, and electrical stimulation. Its use in neuronal cultures has led to increases in the length of neurite outgrowth. Experimental evidence suggests that PEDOT shows a low immunogenic response when implanted in a rat cortical cortex. However, neurons have shown a loss of F-actin stress fibers when cultured on PEDOT. Moreover, neurons have been seen to begin to die of apoptosis after one-week post-implantation [129,130]

Many challenges remain to be overcome before using conductive polymers as brain implants [121,131]. Vigorous basic and clinical research must be carried out to develop conductive polymers for electrical stimulation of neurons, with no adverse cytotoxic effects, to generate safe devices for medical applications.

## 4. Materials and Techniques Commonly Used for the Fabrication of Bioscaffolds for Neural Tissue Engineering

Here, we provide a comprehensive summary of the most frequently utilized polymers for scaffold fabrication in brain tissue engineering, facilitating the selection of appropriate materials based on desired scaffold properties (see Table A1, Appendix A). Table A2 outlines the current techniques employed for scaffold fabrication in brain tissue engineering, enabling researchers and practitioners to identify suitable approaches for scaffold production, considering the advantages and disadvantages of each technique, and examples of the materials with which techniques have been applied (see Table A2, Appendix B). The usability of these tables aids in streamlining the decision-making process for scaffold design, ultimately in enhancing the efficiency and effectiveness of brain tissue engineering research and development.

## 5. Future Perspectives

The design of scaffolds for brain tissue production is not trivial. Materials should be scalable with regard to morphology and structure, i.e., pore size, mechanical properties, nano-topography, biochemical cues, electrical properties, etc. The way that cells respond to artificial substrates with determined physicochemical properties is far from being fully understood [132,133].

Incorporating nanotechnology can enable the tailoring of molecular interactions between neurons, glial cells, astrocytes, and scaffolds [134]. Future research should be conducted to understand those interactions in 3D cultures and organoids, due to the better accuracy in replicating the microenvironment of neural tissues instead of traditional 2D cultures that often limit real-life interactions in vivo [135].

Among all fabrication techniques for scaffolds, 3D bioprinting is an emergent and powerful bio-fabrication strategy. Unlike previous techniques, 3D bioprinting offers the possibility of positioning biologics, including living cells and extracellular matrix components, in combination with inert materials. Three-dimensional bioprinting aims to revolutionize the future design of scaffolds for the next generation of brain tissue implant engineering [136,137].

## 6. Conclusions

The urgent need to address global health concerns and replace damaged tissues has propelled the convergence of innovative approaches, designs, and technologies toward restoring functional tissues. In the realm of brain tissue engineering, we have identified two critical challenges demanding our attention and expertise: Firstly, unraveling the intricate cell interactions with nano-topographies, which hold the key to determining cell fate, phenotype, and behavior. Secondly, obtaining a material that meets the stringent requirements of biocompatibility, safety, biodegradability, porosity, topography, controlled substance release, and conductivity while maintaining precise quality control and reproducibility.

In this dynamic context, the construction of scaffolds emerges as a transformative avenue, offering an array of captivating advantages. By embracing this approach, we open doors to developing scalable, reproducible, and inherently safe medical devices that faithfully replicate the natural extracellular matrix environment. With every step forward in scaffold engineering, we propel the boundaries of possibility, inching closer to a future where neurologically impaired individuals can experience restored brain function and renewed hope.

## Figures and Tables

**Table 1:** Immobilized growth factors in scaffolds for neural tissue engineering.

Immobilized Growth Factor	Effect	Substrate	Reference
VEGF	Enhanced angiogenesis and inhibited formation of glial scars at the injured sites	HA	[88]
NGF and FGF-2	Improves extension and infiltration of neurites and provides neurite guidance.	Chitosan films, Polyamide nanofibers.	[89,90]
LIF and SCF	Maintenance of pluripotent state up to two weeks.	Maleic anhydride copolymer thin films.	[91]
BNDF	Triggers pluripotent cell differentiation into specific lineages such as neurons or oligodendrocytes, and improvement in synaptic communication. Enhances neural stem cell proliferation.	3-D electrospun poly-epsilon-caprolactone nanofibers.	[92,93]
TGF-β1	Reduced astrocyte proliferation and glial scar.	Oxidized dextran with sodium metaperiodate.	[94]
GDNF	Increased myelination of regenerating axons.	Positively-charged oligo[poly(ethylene glycol)fumarate].	[95]

VEGF: Vascular endothelial growth factor, NGF: nerve growth factor, FGF-2: fibroblast growth factor 2, LIF: leukemia inhibitor factor, SCF: stem cell factor, BNDF: brain-derived neurotrophic factor; TFG-β: transforming growth factor-beta, GDNF: glial-cell-derived neurotrophic factor.

## Appendix A

**Table A1:** Main polymers used for the design of scaffolds in brain tissue engineering.

Material	Origin	Characteristics	Applications	References
Alginate	Natural	Hydrogel-based biodegradable scaffold.	Axonal regeneration.	[138]
Cellulose	Natural	Hydrogel	Tissue repair, stem cell therapy, anti-inflammatory drug delivery.	[139]
Chitosan	Natural	Hydrogel-based biodegradable scaffold.	Restoration of cell function, axonal regeneration, drugs, and neurotrophic factor release.	[140,141,142]
Collagen	Natural	Hydrogel-based biodegradable scaffold.	Cell survival and proliferation, tissue repair, restoration of cell function, growth factors release, axonal regeneration, and stem cell therapy. To date, this is the only material in clinical trials used for neural tissue engineering.	[143,144]
Nanopeptide hydrogel	Natural	Hydrogel-based biodegradable scaffold.	Cell survival and proliferation, tissue repair, restoration of cell function, angiogenesis.	[145]
Gelatin	Natural	Hydrogel-based biodegradable scaffold.	Cell survival, proliferation, blood–brain barrier restoration, tissue repair, anti-inflammatory properties, and stem cell therapy.	[146,147,148]
HA	Natural	Hydrogel	Cell survival; axonal regeneration, growth factor release; stem cell therapy; promotion of glial, neuronal, or immature/progenitor states; vascularization.	[88,149,150]
Xyloglucan	Natural	Hydrogel	Axonal regeneration, tissue repair, stem cell survival.	[151]
PVA	Synthetic	Hydrogel-based scaffold.	Cell survival and proliferation, controlled drug release.	[152]
PCL	Synthetic	Hydrogel-based biodegradable scaffold.	Axonal regeneration, cell survival, restoration of cell function, neurotrophic factors release, stem cell therapy.	[132,153]
PEG	Synthetic	Hydrogel-based biodegradable scaffold.	Axonal regeneration, reduced microglia and astrocyte response; and neurotrophic factor release.	[154,155]
PHEMA	Synthetic	Hydrogel-based scaffold.	Axonal regeneration, cell survival, growth factor release.	[156]
PHMA	Synthetic	Hydrogel	Axonal regeneration, anti-inflammatory properties.	[157]
PLGA	Synthetic	Hydrogel-based biodegradable scaffold.	Axonal regeneration, vascularization, tissue repair.	[158]
Polyurethan	Synthetic	Hydrogel-based biodegradable scaffold.	Axonal regeneration, cell survival, cell function restoration, and stem cell therapy.	[107,159,160]
PuraMatrix®	Synthetic	Hydrogel-based biodegradable scaffold.	Stem cell therapy.	[161]
Ppy	Synthetic	Conductive scaffold	Axonal regeneration, cell proliferation, drug release, electrical stimulation.	[162,163]
PANi	Synthetic	Conductive scaffold	Axonal regeneration, cell proliferation, drug release, electrical stimulation.	[164]
PEDOT	Synthetic	Conductive scaffold	Axonal regeneration, cell proliferation, drug release, electrical stimulation, and recording.	[129,165]

HA: hyaluronic acid, PCL: Poly-ε-caprolactone, PVA: Poly(vinyl alcohol co-vinyl acetate), PANi: Polyaniline, PEDOT: Poly (3,4-ethylenedioxythiophene), PEG: Poly(ethylene glycol), PHEMA: Poly(2-hydroxyethyl methacrylate), PHMA: Poly(2-hydroxypropyl methacrylate), PLGA: Poly(lactic-co-glycolic acid), Ppy: Polypyrrole.

## Appendix B

**Table A2:** Pros and cons of the main fabrication methods used in the design of scaffolds in brain tissue engineering.

Fabrication Method	Principle	Pros	Cons	Examples of Materials	References
Electrospinning	Electrospinning uses electricity and fluid dynamics to create fibers. It starts by electrifying a liquid droplet, which then forms a jet. This jet is then stretched and elongated to produce one or more fibers.	*Wide material choice. *Nanofibrous architecture that offers benefits for cells. *Simplicity.	*Poor scalability. *Low reproducibility. *Difficulties in creating 3D scaffolds with well-defined pore architecture. *No shapes other than cylinders and sheets are possible.	PLGA, PCL, PEO, PVA, collagen, gelatin, chitosan, silk protein, fibrinogen.	[166,167]
Solvent casting	Solvent casting utilizes the evaporation of certain solvents to create scaffolds in a mold.	*Simple procedure. *Pore size can be controlled using appropriate molds. *Easy incorporation of drugs into the scaffold.	*Use of highly toxic solvents. *Poor pore interconnectivity.	PCL, PLA.	[168]
Soft lithography	Soft lithography is a collection of techniques that involve fabricating or replicating structures using elastomeric stamps, molds, and conformable photomasks.	*Low cost. *High biocompatibility. *High resolution (5 to 100 nm).	*The resolution can be reduced by the diffusion of ink.	GPS, PMMA, soft-gel materials.	[169,170]
Electrospray	Electrospray uses a conductive solvent to create micro and nanoparticles from a polymer solution. Unlike electrospinning, the size and shape of the particles produced can be controlled by adjusting factors such as concentration, solvent choice, and viscosity.	*Wide material choice. *Formation of homogeneous nanoparticles. *Simplicity. *Nanoparticles with high loading capacity.	*Poor scalability. *No shapes other than nano and microspheres.	Chitosan.	[171]
Porogen leaching	Porogen leaching entails casting a solution of polymer and porogen into a mold, drying the mixture, evaporating the solvent, and then leaching the embedded porogen with water to create pores.	*Control over porosity and pore geometry. *Low-cost.	*Inadequate pore size and interconnectivity.	PLA, alginate, gelatin.	[172]
Self-assembly	A natural arrangement of molecules, where disordered entities organize into ordered structures due to specific interactions (van der Waals forces, hydrophobic, electrostatic, hydrogen bonding, π–π aromatic stacking, metal coordination, etc.) among the components.	*Control over porosity and fiber diameter. *Regular structures are obtained. *Simple and versatile strategy. *Mimics natural structures.	*Minimal control over the shape. *Limited use of materials.	Silk, liposomes, VLP, DNA.	[173,174]
Bioprinting	Bioprinting is an additive manufacturing technique involving layer-by-layer printing of living cells using bioinks. This process aims to create structures that mimic the behavior and structures found in natural tissues.	*Offers flexibility, customization, scalability, reliability, durability, and relatively high speed. *Enables the design of both 2D and 3D structures.	*Expensive *Limited cell viability.*Poor reproducibility and scalability. *It is challenging to recreate the intricate microarchitecture and vascular networks.	PCL, PLA, PLGA, PEO/PBT, pluronic, GelMA, HA, PVA.	[175]
Microcontact printing	Microcontact printing involves creating a stamp or mold with the desired pattern or features on its surface. The stamp is then inked or coated with a material, such as an ink or self-assembled monolayer, which can adhere to the substrate.	*Enables the patterning of proteins of interest on substrates. *Low-cost.	*Poor reproducibility and scalability. *It is not possible to print different molecules with one stamp. *It is difficult to control the amount of protein transferred.	PVA, polyacrylamide, GelMA, graphene.	[176]
Gas foaming	Gas foaming utilizes gas, such as carbon dioxide, as a porogen instead of a solvent.	*Generates highly porous scaffolds. *Low-cost. *Eliminaes the need for harsh chemical solvents.	*Limited mechanical properties. *Inadequate pore interconnectivity. *Poor control of pore size.	PLGA, PEG.	[177,178]

PLGA: Poly(lactic-co-glycolic acid), PCL: Poly-εcaprolactone PEO: Poly(ethylene oxide), PVA: Poly(vinyl alcohol), PLA: Poly(D,L-lactide), GPS: Graphene oxide-based patterned substrate, PMMA: Poly(methyl methacrylate), VLP: Virus-like particles, PEO/PBT: Poly(ethylene oxide terephthalate-*co*-butylene terephthalate, HA: Hyaluronic acid, GelMA: Gelatin methacrylate, PEG: Poly(ethylene glycol).
